# Essential Functions of the Histone Demethylase Lid

**DOI:** 10.1371/journal.pgen.1001221

**Published:** 2010-11-24

**Authors:** Ling Li, Christina Greer, Robert N. Eisenman, Julie Secombe

**Affiliations:** 1 Division of Basic Sciences, Fred Hutchinson Cancer Research Center, Seattle, Washington, United States of America; 2 Department of Genetics, Albert Einstein College of Medicine, Bronx, New York, United States of America; Max-Planck-Institute of Immunobiology, Germany

## Abstract

Drosophila Little imaginal discs (Lid) is a recently described member of the JmjC domain class of histone demethylases that specifically targets trimethylated histone H3 lysine 4 (H3K4me3). To understand its biological function, we have utilized a series of Lid deletions and point mutations to assess the role that each domain plays in histone demethylation, in animal viability, and in cell growth mediated by the transcription factor dMyc. Strikingly, we find that *lid* mutants are rescued to adulthood by either wildtype or enzymatically inactive Lid expressed under the control of its endogenous promoter, demonstrating that Lid's demethylase activity is not essential for development. In contrast, ubiquitous expression of UAS-Lid transgenes lacking its JmjN, C-terminal PHD domain, and C_5_HC_2_ zinc finger were unable to rescue *lid* homozygous mutants, indicating that these domains carry out Lid's essential developmental functions. Although Lid-dependent demethylase activity is not essential, dynamic removal of H3K4me3 may still be an important component of development, as we have observed a genetic interaction between *lid* and another H3K4me3 demethylase, *dKDM2*. We also show that Lid's essential C-terminal PHD finger binds specifically to di- and trimethylated H3K4 and that this activity is required for Lid to function in dMyc-induced cell growth. Taken together, our findings highlight the importance of Lid function in the regulated removal and recognition of H3K4me3 during development.

## Author Summary

Correct spatial and temporal control of gene expression is essential for development. One of the many ways that gene expression is regulated is by the addition, recognition, and removal of methyl groups from the histone proteins around which DNA is wrapped within the nucleus. Here we describe a systematic analysis of Little imaginal discs (Lid), a protein that regulates transcription via a number of different mechanisms that involve regulated removal and recognition of histone methylation. We show that while Lid's histone demethylase activity is not essential for development, numerous other conserved domains of this protein are. Furthermore, we find a genetic interaction between *lid* and another histone demethylase, *dKDM2*, that suggests this enzyme can compensate for the loss of Lid's enzymatic activity. These findings have significance for our insight into how gene expression is normally regulated and have implications for our understanding of how this goes awry during disease progression.

## Introduction

The Drosophila *lid* gene is essential for development and encodes a protein with multiple domains implicated in chromatin-mediated regulation of transcription, including the recently described lysine demethylase domain, Jumonji C (JmjC). Six lysine residues of histones H3 and H4 can be mono, di or trimethylated, and each modification is found in a stereotypical pattern with respect to the coding region of a gene and correlates with a different transcriptional outcome [1]–[4]. As a general rule, methylation of H3K4, K36 or K79 is found at active genes whereas H3K9, K27 and H4K20 methylation is associated with those that are repressed. We and others have shown that overexpression of Lid reduces H3K4me3 levels and that this chromatin mark is elevated in *lid* mutants, establishing Lid as a JmjC domain-dependent H3K4me3 demethylase [5]–[7]. The four conserved mammalian orthologs of Lid, KDM5a-d, also demethylate H3K4me3 although these proteins show broader substrate specificity than their Drosophila counterpart, also targeting H3K4me2 [8]–[10].

While there is limited data regarding the biological role of the KDM5 family of proteins in mammals, the findings that KDM5b is overexpressed in breast, bladder and prostate cancers [11]–[13] and that mutations in KDM5c are found in patients with X-linked mental retardation [14] suggest that they play important developmental roles. However, a confounding factor to the analysis of the four mammalian KDM5 paralogs is their functional redundancy, as the mouse KDM5a knock out is viable, fertile and displays no change in global H3K4me2/3 levels. In contrast, Lid is the sole KDM5 protein in Drosophila and it is essential for viability [15], providing an ideal system in which to investigate the function of this family of proteins.

Although the KDM5 family of proteins are named based on the function of their catalytic JmjC domain, metazoan KDM5 proteins have several other conserved motifs: a JmjN domain of unknown function that is present in a subset of JmjC proteins, an ARID (A/T rich interaction domain [16]) implicated in binding both A/T and G/C rich DNA sequences [17], [18], a single C_5_HC_2_ zinc finger, and two or three PHD fingers (plant homeobox domain [19]) involved in mediating protein-protein interactions [20]. Importantly, while the JmjC domain-dependent demethylase function is well defined in vitro for KDM5 family proteins, the in vivo relevance of this and other domains remains unclear.

We have previously shown that Lid is rate-limiting for cell growth induced by the Drosophila homolog of the c-Myc oncoprotein, dMyc [7]. Specifically, Lid binds directly to dMyc and is required for dMyc-dependent activation of one of its growth regulatory target genes, *Nop60B*. While we have demonstrated that this occurs independently of Lid's lysine demethylase activity, the molecular mechanism by which Lid functions in Myc-mediated growth is yet to be determined. Here we present an investigation of the function of Lid's domains and demonstrate that its demethylase activity is dispensable for development, however its JmjN, PHD3 and C_5_HC_2_ domains are all essential. While our observation that Lid's demethylase activity is not essential suggests that regulated removal of H3K4me3 serves primarily to modulate gene expression levels, a genetic interaction between *lid* and the JmjC domain-containing protein *dKDM2* is consistent with these two demethylases acting redundantly on H3K4me3. We also show that the essential C-terminal PHD finger of Lid binds di- and trimethylated H3K4 and that this domain is required for *lid* to genetically interact with dMyc. Based on these data, we propose that Lid-dependent recognition of H3K4me2/3 facilitates dMyc binding to promoters rich in this active chromatin mark.

## Results

### The JmjN, ARID, PHD1, and C_5_HC_2_ zinc fingers of Lid are required for demethylase activity

To assess the contribution of each individual domain of Lid to its demethylase activity and animal development, we generated a series of deletions and point mutations that disrupt each domain of Lid to complement our previously characterized demethylase inactive version of Lid (Lid-JmjC*) that harbors two point mutations in the JmjC domain and prevents Fe^2+^ binding (H637 and E639 to Alanine) [7]. To enable these analyses, flies carrying UAS transgenes that specifically delete Lid's JmjN, ARID, C_5_HC_2_ zinc finger and three PHD fingers were generated to allow conditional Gal4-mediated expression in vivo (see Materials and Methods for details).

**Figure 1:**
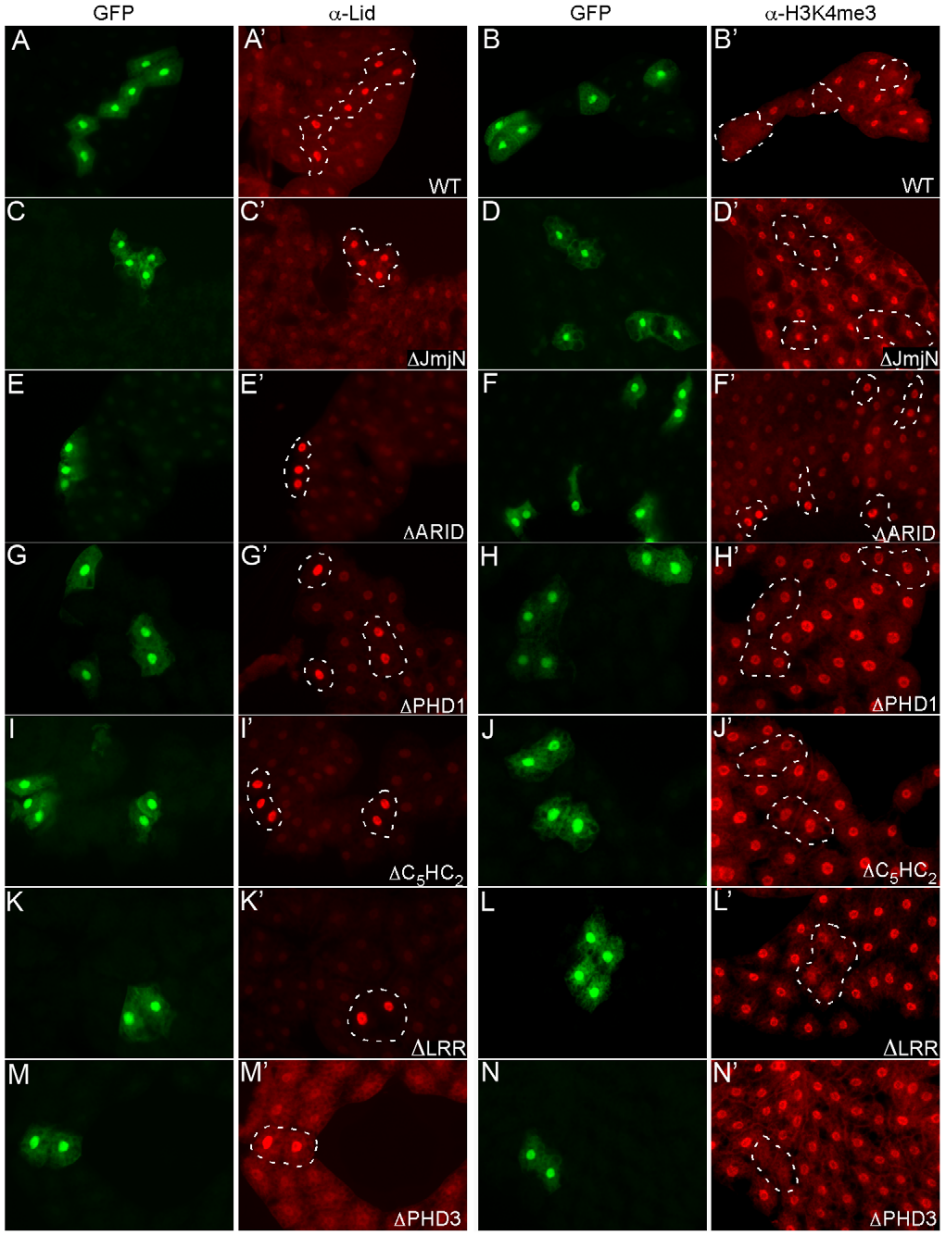
Deletion of Lid's JmjN, ARID, and PHD1 domains abrogate its demethylase activity. Clones of cells expressing Lid or Lid mutant transgenes in fat body were generated by crossing hs-FLP; UAS-Lid females to act>CD2>Gal4, UAS-GFP males. No heat shock was carried out since leaky FLP expression during embryogenesis leads to a small number of fat body clones. Levels of Lid (A', C', E', G', I', K', M') and trimethylated H3K4 (B', D', F', H', J', L', N') were examined. Cells expressing each transgene (as labeled on figure) are marked by co-expression of GFP and are outlined in the other panels.

To assess the ability of our Lid mutants to demethylate, we generated clones of cells overexpressing each protein in larval fat body and examined the levels of Lid and H3K4me3 (Figure 1). Based on the intensity of the immunofluorescence signal and Western analysis, all of our transgenes expressed Lid at similar levels (data not shown; Figure 1), with the exception of Lid^ΔPHD2^, for which we were unable to detect Lid overexpression even after combining multiple transgenes (data not shown). To examine the role of Lid's second PHD finger, we created a point mutant in the first cysteine of this C_4_HC_3_ zinc finger (UAS-Lid^C1296A^) and found that overexpression could be detected after combining two transgenes (Figure 1K). Mutating the second or deleting the third PHD domain of Lid did not affect its ability to demethylate H3K4me3 (Figure 1K–1N). In contrast, Lid's JmjN, PHD1 or C_5_HC_2_ domains were essential for enzymatic activity as overexpression of these deletion mutants resulted in no change in global levels of H3K4me3 (Figure 1D, 1H, 1J). While the role of Lid's PHD1 and C_5_HC_2_ domains in demethylation remains to be investigated, our finding that Lid's JmjN domain is required for demethylase activity is not surprising based on structural analysis of the demethylase KDM4a which shows its JmjN domain making extensive contacts within the catalytic core of its immediately adjacent JmjC domain [21]. Unlike other deletions that prevented Lid's enzymatic function, expression of Lid^ΔARID^ resulted in a variable increase in H3K4me3 levels, indicating that this mutant protein can behave as a dominant negative in fat body cells (Figure 1E, 1F). We do not yet understand the mechanism by which Lid^ΔARID^ increases H3K4me3 levels, but have observed a similar effect upon overexpression of Lid-JmjC* [7]. The ARID of KDM5a, b and Lid are required for demethylase activity in transient transfection assays, however a dominant interfering effect has not been reported [17], [22]. Our finding that deletion of Lid's ARID can increase H3K4 trimethylation raises the possibility that in addition or concomitant with its ability to bind DNA, this domain may cooperate with Lid's JmjC domain.

### Lid's demethylase activity is dispensable for development

**Figure 2:**
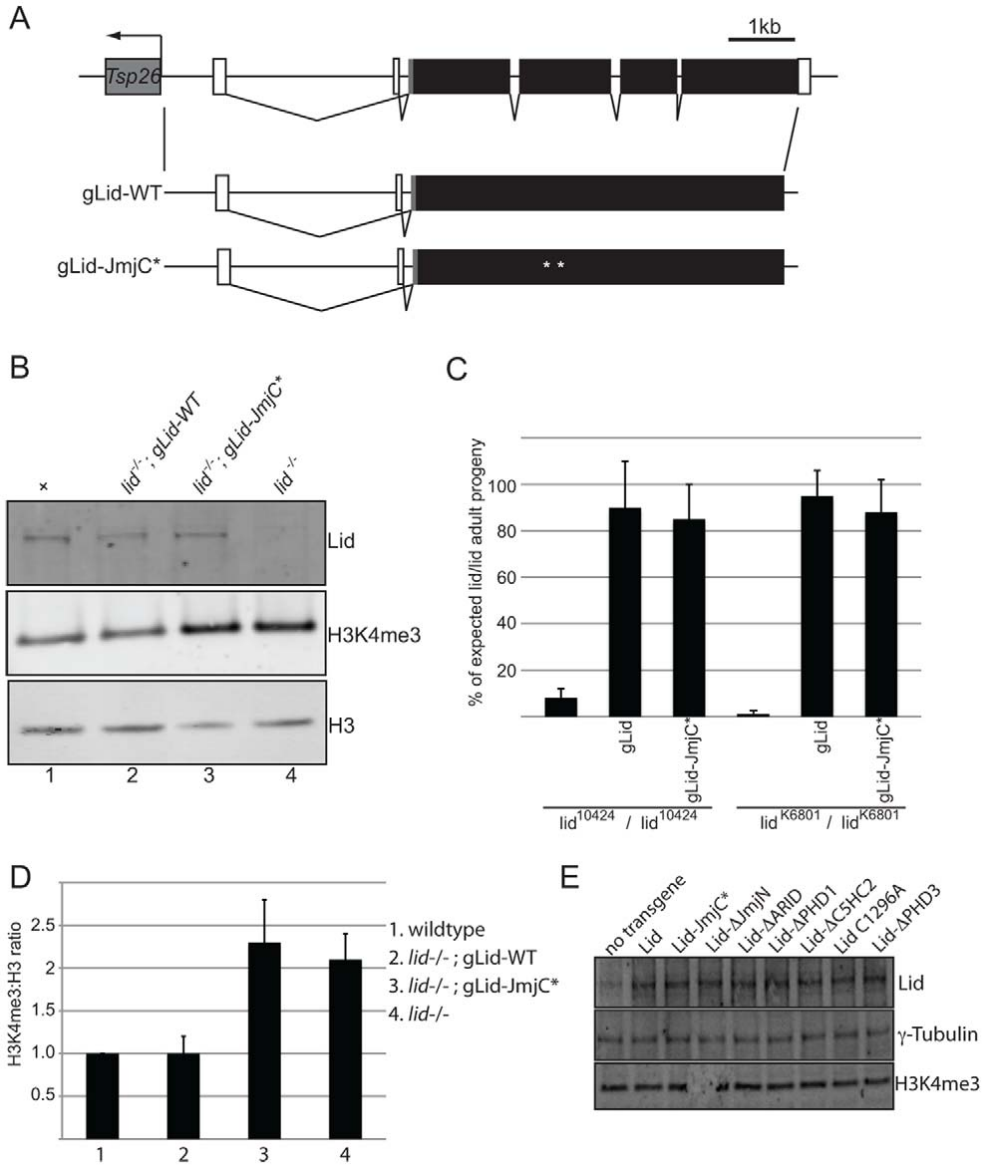
Wildtype and demethylase inactive genomic rescue transgenes can rescue *lid* mutant animals. (A) Schematic representation of wildtype and mutant rescue transgenes in which 4.6 kb of upstream regulatory region fused to a Lid cDNA. (B) Western blot analysis of wing discs using anti-Lid, anti-H3K4me3 and anti-histone H3 in wildtype (lane 1), *lid/lid*; gLid-WT, (lane2), *lid/lid*; gLid-JmjC* (lane 3) and *lid^10424^* homozygous (*lid/lid*, lane 4). (C) Quantitation of rescue based on the % of each genotype expected based on Mendelian genetics from the cross of *lid^10424^*/CyO (or *lid^K6801^*/CyO) females to *lid^10424^*/CyO; gLid-WT or gLid-JmjC* males in uncrowded (10 females per cross) conditions. (D) Quantitation of H3K4me3:H3 ratios from three independent experiments described in (B). (E) Western analysis of wing discs from Actin-Gal4 based rescue crosses. 12 wing discs were used per lane and the Western probed with anti-Lid, anti-H3K4me3 and the loading control γ-tubulin.

To determine the importance of Lid's conserved domains in vivo, we ubiquitously expressed our UAS-Lid transgenes in animals lacking endogenous zygotic *lid* expression. Because Lid is normally expressed ubiquitously throughout development [7](data not shown), we expressed our UAS-Lid (and Lid mutant) transgenes at low uniform levels in *lid^10424^* homozygous mutants using actin-Gal4 (Figure 2E). This approximately two-fold overexpression of Lid is not sufficient to cause any change to global levels of H3K4me3 in wing discs (Figure 2E). As a control, we crossed our Lid transgenes to actin-Gal4 in a wildtype background to ensure that expression of these Lid mutants did not have any deleterious effects. In all cases, expression of our UAS transgenes in a wildtype background gave viable adults, however UAS-Lid^ΔARID^, Lid-JmjC* or Lid^ΔPHD1^ expressing adult females failed to lay eggs, so were sterile. Surprisingly, ovaries from females expressing these three mutant forms of Lid were phenotypically normal as assessed by dapi and phalloidin staining (data not shown), so the basis for their dominant interference with oviposition is not clear. This effect on egg laying is likely to be due to expression these Lid mutant transgenes in somatic cells of the ovary since germline specific expression using Nanos-Gal4 does not result in sterility (data not shown).

As expected, actin-Gal4 driven expression of wildtype Lid rescued *lid^10424^* mutant animals at the expected Mendelian frequency (Table 1). In contrast, expression of UAS-Lid harboring deletions of its JmjN, ARID, or PHD3 domains fail to rescue *lid* mutants, suggesting that these domains are essential for development. Expression of Lid^ΔC5HC2^ resulted in a small percentage (29%) of *lid* mutant flies eclosing, all of which died within several days indicating that this domain is essential in adults. In contrast to the third PHD, we found that the first and second PHD fingers of Lid are dispensable for development. While both sexes rescued by Lid^C1296A^ were fertile, Lid^ΔPHD1^-rescued females were sterile and, like overexpression of this transgene in a wildtype background, Lid^ΔPHD1^-rescued flies had phenotypically normal ovaries. We also tested our previously generated JmjC domain point mutant that abolishes demethylase activity for rescue of *lid*-associated lethality. Actin-Gal4-mediated expression of Lid-JmjC* failed to rescue *lid* mutants (Table 1), initially suggesting that Lid's demethylase activity is essential for development. However, since overexpression of Lid-JmjC* behaves as a dominant negative in a tissue specific manner, most notably in larval fat body cells [7], it may interfere with maternally deposited wildtype Lid in the rescue experiments described above.

**Table 1:** Domains of Lid that are essential for development.

	% of expected progeny for genotype listed		
Strain crossed to *lid^10424^*/*CyO*; Actin-Gal4/*TM3*	*lid^10424^/lid^10424^, UAS-lid (orΔ)*; actin-Gal4/+	*lid^10424^/lid^10424^, UAS-lid (orΔ)*; *TM3*/+	*Total flies scored*
*lid^10424^/CyO*	1%	0.5%	429
*lid^10424^,* UAS-lid*/CyO*	82%*	0.5%	169
*lid^10424^,* UAS-lid-JmjC* */CyO*	0%	0%	110
*lid^10424^,* UAS-lidΔJmjN*/CyO;* UAS-lidΔJmjN	2%	0%	107
*lid^10424^/CyO;* UAS-lidΔARID	0%	0%	160
*lid^10424^,* UAS-lidΔPHD1*/CyO*	88%*	0.1%	198
*lid^10424^,* UAS-lidΔC_5_HC_2_ */CyO*	29%$ *	0%	154
*lid^10424^,* UAS-lidC1296A*/CyO*; UAS-LidC1296A	100%*	2%	95
*lid^10424^,* UAS-lidΔPHD3*/CyO*	0.5%	0%	337

Rescue of *lid^10424^* by Lid deletion transgenes: 7 female flies of the genotype shown in left column were crossed to *lid^10424^*/CyO; Actin-Gal4/TM3 at 25°C and the progeny scored. Genotypes not shown were obtained at the expected Mendelian frequency.

*indicates that the number of *lid^10424^*/*lid^10424^* UAS-Lid transgene; actin-Gal4 flies obtained were significantly more than control crosses lacking the transgene (chi squared test).

$ All flies of this genotype died within several days of eclosing.

To address the function of Lid's demethylase activity during development, we therefore generated genomic rescue transgenes that fused 3.9 kb of Lid's upstream regulatory region to either a wildtype or JmjC* mutant form of the *lid* coding region (gLid-WT and gLid-JmjC* respectively; Figure 2A). gLid-WT and gLid-JmjC* transgenes were then crossed into *lid^10424^* and *lid^k6801^* mutant backgrounds, the levels of transgene expression confirmed, and the number of homozygous *lid* mutant flies scored (Figure 2B, 2C; data not shown). Strikingly, *lid* mutant animals carrying one or two copies of a gLid-WT or gLid-JmjC* transgene produced phenotypically normal and fertile adult flies at the predicted frequency (Figure 2C; data not shown). Lid's demethylase activity is therefore not essential for Drosophila development. Based on the rescue of *lid* mutants by enzymatically inactive Lid expressed at endogenous levels, it is likely that this mutant form of Lid failed to rescue in our actin-Gal4 based rescue experiments because its overexpression interferes with maternally deposited wildtype Lid. It is therefore possible that Lid^ΔARID^ also fails to rescue *lid* mutants due to its dominant interference with endogenous Lid, thus further examination of this domain will require generation of transgenes using *lid*'s endogenous promoter.

### Demethylase inactive animals have increased levels of H3K4me3

A majority of homozygous *lid* mutant animals die during pupal development and have increased global levels of H3K4me3 [5], [7], [22], [23]. While Lid can remove di and trimethylated histone H3K4 peptides in vitro, we and others have shown that it only targets H3K4me3 in vivo as only this methyl mark is altered upon Lid overexpression, in *lid* mutants, and in response to Lid RNAi [5], [7], [22]. Because expression of the demethylase inactive form of Lid is able to rescue *lid* mutants, we asked whether these animals also have increased global levels of H3K4me3. To examine this, we dissected wing discs from wildtype and *lid^10424^* homozygous mutant larvae and compared the levels of H3K4me3 to *lid* mutants carrying two copies of gLid-WT or gLid-JmjC* by Western blot. As seen in Figure 2B and 2D, gLid-JmjC* animals show increased H3K4me3 indistinguishable from that observed in *lid* mutants, demonstrating that the increased level of H3K4me3 observed in *lid* mutant animals is not the cause of their lethality.

### Demethylase inactive Lid flies are short-lived

**Figure 3:**
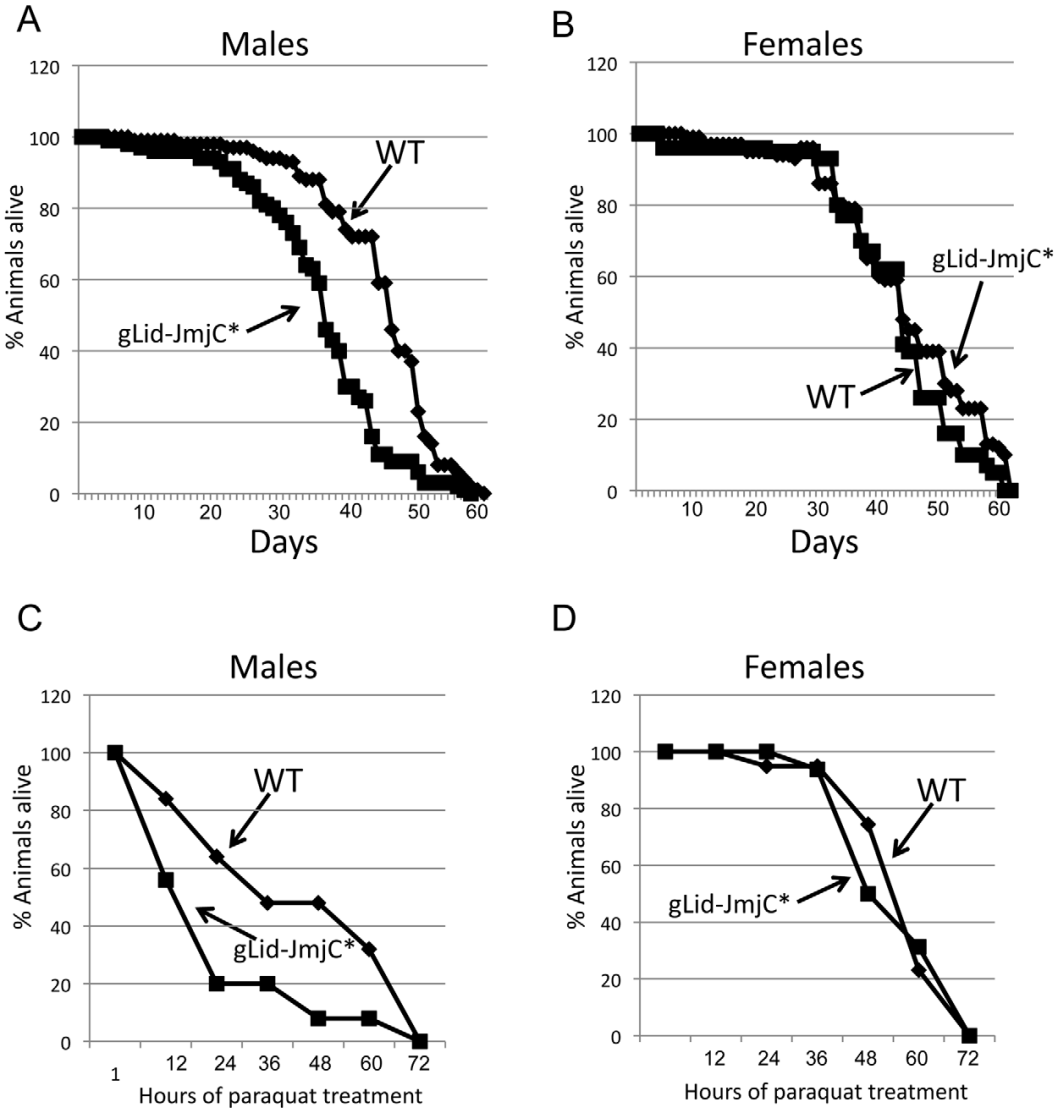
Demethylase inactive male flies are short-lived and sensitive to paraquat. (A, B) Lifespan of wildtype and demethylase inactive Lid males and females, respectively showing that males are short-lived whereas females have a normal lifespan. (C, D). Survival in response to 20 mM paraquat of wildtype and demethylase inactive males and females, respectively. Like lifespan, males are sensitive to paraquat whereas females are not.

Mutants and RNAi-mediated knock-down of the C. elegans Lid ortholog RBR-2 result in elevated levels of H3K4me3 and a 15–25% reduction in lifespan [24]. To determine whether this is a conserved, demethylase-specific phenotype, we assessed the lifespan of our demethylase inactive Lid flies. We find that *lid* mutant males rescued by gLid-JmjC* have a significantly shorter lifespan (mean of 37 days) than their wildtype (45 days) or gLid-rescued (46 days) flies (Figure 3A; data not shown). Interestingly, this effect is not observed in females, with the average lifespan of gLid-JmjC*-rescued flies not being significantly different to wildtype (48.1 and 45.1, respectively; Figure 3B). Lacking H3K4me3 demethylase activity therefore has adverse effects on processes required during male adulthood and suggests lifespan phenotypes observed in *C. elegans* hermaphrodites are likely to be specific to modulation of H3K4me3 levels rather than other RBR-2-dependent processes.

Animals with reduced life expectancies also often show sensitivity to oxidative stressed induced by paraquat. We therefore treated wildtype and demethylase inactive flies with paraquat and found a sex-specific effect of this inducer of oxidative damage. In a similar manner to our lifespan studies in which males were more dramatically affected than females, we find that males are sensitive to paraquat whereas females are not (Figure 3C, 3D). Male Drosophila are therefore more sensitive to the loss of Lid-dependent H3K4me3 demethylation than females, although the molecular basis for this remains unclear.

### The JmjC domain-containing demethylases Lid and dKDM2 genetically interact

One explanation for our finding that the loss of Lid's enzymatic activity does not adversely affect development is that its H3K4me3 demethylase activity is compensated for by another demethylase. To date, the JmjC domain-containing protein dKDM2 is the only other Drosophila protein shown to target H3K4me3, although it has also been reported to remove H3K36me2 [25], [26]. To address whether dKDM2 and Lid act in a redundant manner, we tested whether hypomorphic mutations in these two genes genetically interact. *lid^K6801^* homozygotes survive until adulthood at a very low frequency (0.5%), but reach pupal development at 71% of the expected frequency (Table 2). The strongest dKDM2 allele, *dKDM2^DG18120^*, is semi-lethal with homozygous adults eclosing at 62% of the expected frequency (Table 2) and these adults are phenotypically normal and fertile. By combining these two mutations, we have found that the phenotype of *lid*, *dKDM2* double mutants is significantly stronger than either single mutant (Table 2), with animals dying during the 1^st^ and 2^nd^ larval instar stages. To demonstrate that Lid's demethylase activity is required for this genetic interaction, we tested whether *lid*;*dKDM2* double mutants could be rescued by our gLid-WT or gLid-JmjC* genomic rescue transgenes. As shown in Table 2, gLid-WT, but not gLid-JmjC* rescued *lid*;*dKDM2* animals, suggesting that Lid and dKDM2 act redundantly in the regulation of H3K4me3.

**Table 2:** *lid* and *dKDM2* genetically interact.

	% of expected progeny for genotype listed	
Genotype	3^rd^ instar larvae	Adults
*+/+; dKDM2^DG18120^/dKDM2^DG18120^*	58%	62%
*lid^K6801^/lid^K6801^; +/+*	71%	0%
*lid^K6801^/lid^K6801^; dKDM2^DG18120^/dKDM2^DG18120^*	0%	0%
*lid^K6801^/lid^K6801^; gLid, dKDM2^DG18120^/dKDM2^DG18120^*	nd	75%
lid^K6801^/lid^K6801^; gLid-JmjC*, dKDM2^DG18120^/dKDM2^DG18120^	nd	0%

*dKDM2* homozygous mutants survive until adulthood at 62% of expected frequency and a majority of *lid^K6801^* homozygotes die during pupal development. In contrast, animals homozygous for *dKDM2* and *lid^K6801^* die during the 1^st^ and 2^nd^ larval instar stages. The lethality associated with *lid*, *dKDM2* double mutants can be rescued by a wildtype, but not a demethylase inactive, genomic rescue transgene.

### The third PHD domain of Lid is required for it to function in Myc-dependent cell growth

**Figure 4:**
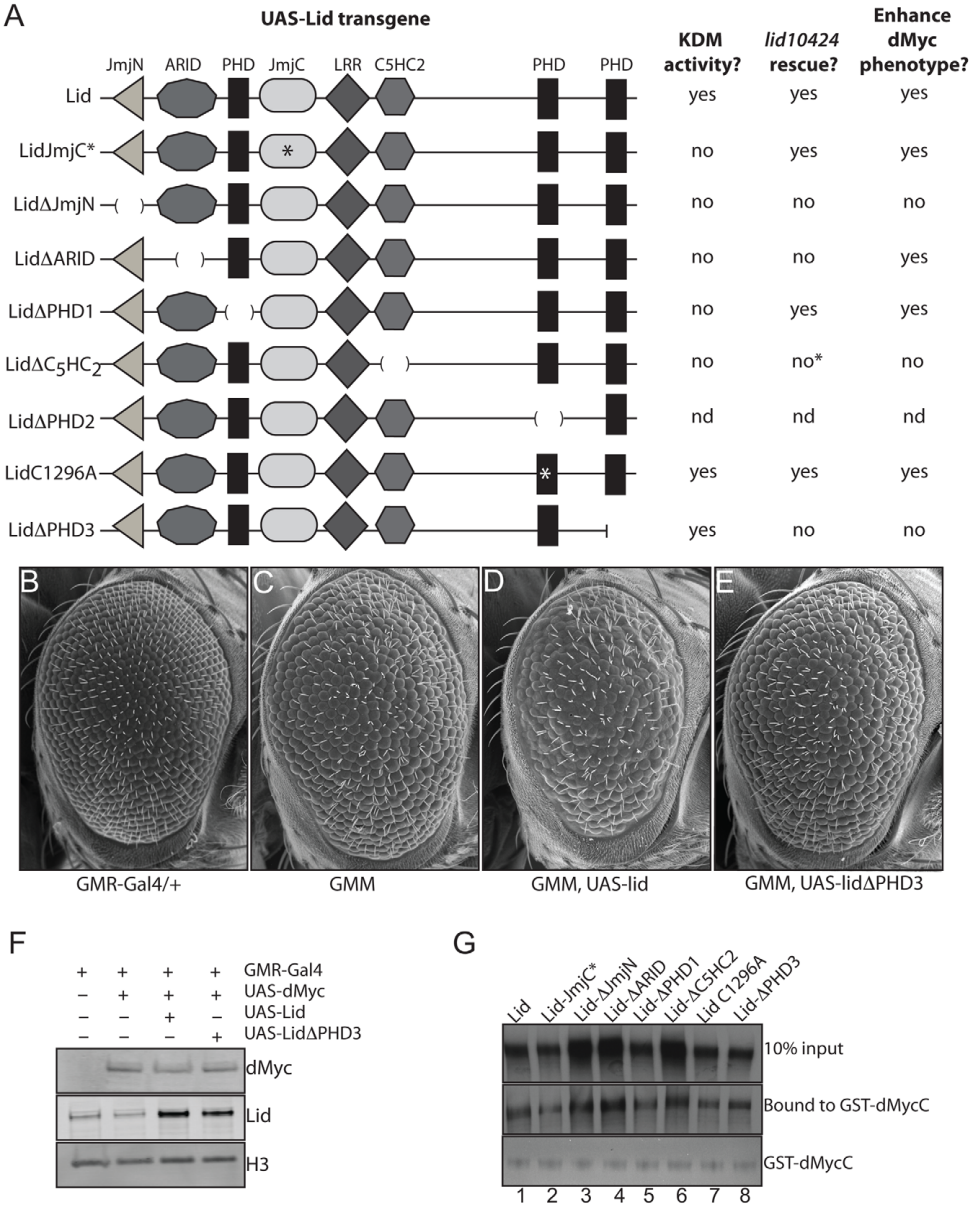
The third PHD finger of Lid is required for it to function with dMyc. (A) Schematic representation of UAS Lid deletion and point mutant transgenes (domains are not shown to scale) summarizing their histone demethylation activity, rescue of lid mutants (*lid^10424^*), and their ability to enhance the dMyc overexpression eye phenotype. (B–E) Scanning electron micrographs of GMR-Gal4 alone (B), GMR-Gal4, UAS-dMyc (3 copies of transgene; GMM) (C), GMM, UAS-Lid (D) and GMM, UAS-LidΔPHD3 (E). B through E all contain 3 copies of the UAS-dMyc transgene. (F) Western analysis of GMR-Gal4 alone (lane 1), GMM (lane 2), GMM, UAS-Lid (lane 3) and GMM, UAS-LidΔPHD3 (lane 4). (G) In vitro binding assays demonstrating that GST-dMyc binds full-length Lid, Lid-JmjC*, LidΔJmjN, LidΔARID, LidΔPHD1, LidΔC5HC2, Lid^C1296A^ and LidΔPHD3 (lanes 1–8, respectively).

We originally isolated *lid* in a genetic screen for regulators and mediators of dMyc-dependent cell growth based on an adult eye phenotype generated by dMyc expression in post-mitotic cells of the developing eye using GMR-Gal4 [7]. Furthermore, we showed that Lid's demethylase activity was not required for its dMyc-dependent functions. To pursue the mechanism by which Lid functions in cell growth induced by dMyc, we crossed our UAS-Lid mutant transgenes to the dMyc overexpressing fly strain and compared their ability to enhance this eye phenotype to that observed in response to expression of wildtype Lid (Figure 4A). Expression of Lid lacking its JmjN, C_5_HC_2_ or PHD3 domains failed to enhance the dMyc overexpression eye phenotype while not altering the levels of overexpressed dMyc (Figure 4B–4F; data not shown). As controls, we expressed the Lid deletion transgenes in a wildtype background and found that they resulted in no adult eye phenotype and, unlike fat body cells, Lid-JmjC* and Lid^ΔARID^ do not have a dominant negative effects in post mitotic cells of the developing eye. We have previously shown that dMyc binds to two regions of Lid: its JmjC domain and its C_5_HC_2_ zinc finger [7]. To verify that all Lid deletion proteins retain their ability to bind dMyc, we carried out in vitro binding assays and found that they all bind equivalently (Figure 4G), suggesting that the JmjN, C_5_HC_2_ and PHD3 domains of Lid are likely to be required for its Myc-dependent functions in cell growth.

### The PHD fingers of Lid bind N-terminal histone tails

**Figure 5:**
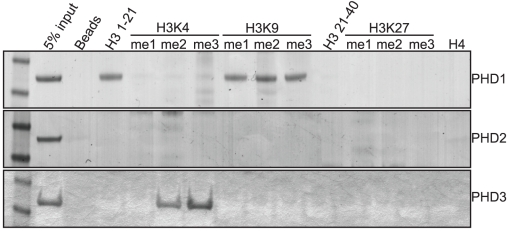
The PHD fingers of Lid bind specific forms of methylated histone tails. GST fusion proteins of Lid's PHD1 (top panel), PHD2 (2^nd^ panel) and PHD3 (3^rd^ panel) were tested for binding to biotinylated histone peptides of histone H3 amino acids 1–21, 21–44 and histone H4 1–21. In addition, peptides mono, di or tri methylated at histone H3 K4, K9 and K27 were also tested for their ability to bind these PHD fingers.

The primary characterized function of Lid is its histone H3 lysine 4 demethylase activity. However, since we have demonstrated that this activity is not Lid's essential function, we chose to further characterize Lid's third PHD finger as this domain is required for it to function with dMyc and is essential for development. Moreover, PHD domains have recently emerged as important interpreters the histone code that act by binding to histone tails that are unmodified, mono-, di- or tri- methylated at specific lysine residues [20]. To address whether Lid's third PHD finger is able to bind methylated histones, we incubated bacterially expressed and purified GST-PHD finger proteins with biotinylated histone peptides mono-, di- or trimethylated at K4, K9 or K27 in vitro and compared this to the binding of Lid's other two PHD fingers and the known H3K4me2/3 binding protein hING2 [27], [28](Figure 5; data not shown).

As seen in Figure 5, Lid's PHD1 finger binds to amino acids 1–21 of histone H3, but not amino acids 21–40 or to histone H4. Lid's PHD1 specifically recognizes unmethylated histone H3 (H3K4me0), as binding is abrogated by mono-, di- or trimethylation of lysine 4, but not methylation of lysine 9. We were unable to detect any in vitro histone binding for Lid's PHD2 finger, however PHD3 bound to both H3K4me2 and H3K4me3, showing a consistent preference for the trimethylated form. The function of both of these PHD fingers is likely to be a highly conserved function of KDM5 proteins, as identical binding specificities have recently been reported for KDM5a [29]. Consistent with the binding of PHD3 to H3K4me2/3 being physiologically relevant, a correlation between KDM5a binding and the presence of this activating chromatin mark has been observed previously using genome-wide arrays, although its physiological relevance has remained elusive [30], [31]. Significantly, the binding of c-Myc also correlates with regions rich in H3K4me2/3 [32]. Based on our findings that Lid's third PHD finger binds H3K4me2/3 and that deleting this domain abolishes its ability to genetically interact with dMyc, we propose that Lid functions to recruit dMyc to regions with high levels of H3K4me2/3 by specifically recognizing this local chromatin context.

## Discussion

Our analyses provide the first investigation of the developmental role of a JmjC domain-dependent demethylase. Five major findings come from this work: (1) Lid's JmjC domain-encoded demethylase activity is dispensable for normal development (2) Loss of Lid's demethylase activity is compensated for by dKDM2 (3) Essential functions of Lid are encoded by its JmjN, C_5_HC_2_ and C-terminal PHD zinc finger motifs (4) The N- and C-terminal PHD fingers of Lid bind specific methylated forms of histone tails (5) Lid's C-terminal H3K4me2/3 binding PHD finger is required for it to function in dMyc-mediated cell growth. Taken together, this significantly extends our knowledge of the role of regulated removal and recognition of di- and trimethylated histone H3 lysine 4 during development.

### Lid-dependent regulation of H3K4me3

Our finding that Lid's lysine demethylase activity is dispensable for development demonstrates that globally increasing the levels of H3K4me3 is not generally detrimental to development. Similarly, elevating H3K4me1/2 levels by mutating the Drosophila demethylase Lsd1 does not adversely affect development, although these animals show some adult phenotypes and subtle changes to expression of the homeobox genes *Ubx* and *Abd-A* [33], [34]. Likewise, Lid's enzymatic activity may serve to fine-tune some gene expression patterns. To date, three genes, *E(spl)m4*, *m7* and *m8*, have been described as direct Lid targets in Drosophila cultured S2 cells, and these show a 4-fold derepression in response to *lid* RNAi and a concomitant increase in promoter-proximal H3K4me3 levels [35]. Furthermore, a genetic interaction has been observed during wing development between *lid* and the *E(spl)* gene upstream regulator *Notch*, suggesting that this regulation is biologically important [35], [36]. In mammalian cells, *MFN2* and *Deltex* expression are repressed upon KDM5a overexpresion, derepressed when KDM5 is knocked-down, and show changes in H3K4me3 levels in their promoters [31], [36]. We examined the levels of *E(spl)m4*, *m7* and *m8*, *Marf1* (the Drosophila ortholog of MFN2) and *Deltex*, but found that their levels were unaltered in RNA extracts from whole larvae or dissected wing imaginal discs from wildtype, *lid* mutant or *lid* mutants rescued by gLid or gLid-JmjC* (JS, unpublished). These genes may therefore be regulated by Lid in a small subset of cells in vivo, so cannot be detected using whole wing disc extracts. Effects on gene expression may also be sex-specific since male flies lacking Lid-dependent demethylase activity have a shortened lifespan and are sensitive to paraquat, whereas females are not.

While removing Lid's demethylase activity does not result in lethality, removing this function in combination with another JmjC domain-containing protein, dKDM2, does. This suggests that in the absence of Lid's demethylase activity, dKDM2 can carry out its essential functions and vice versa. RNAi-mediated knock down of *dKDM2* has been found to increase H3K36me2 levels in S2 Drosophila tissue culture cells and H3K4me3 levels in adult flies [25], [26]. Surprisingly, we find that global levels of H3K4me3 and H3K36me2 are both unchanged in *dKDM2^DG12810^*, *dKDM2^KG04325^* or *dKDM2^EY01336^* homozygous mutant wing discs (CG and JS, unpublished). The reason for the disparity between our results obtained with dKDM2 mutants and previously published data are not clear, but may be due to the difference between the acute loss of dKDM2 mediated by RNAi and the chronic loss in *dKDM2* mutants, or to off target effects of the RNAi. The most characterized function of dKDM2 and its mammalian orthologs (KDM2A, KDM2B) is its regulation of rRNA expression [25], [37]. Interestingly, repression of rRNA transcription by KDM5A correlates with changes to H3K63me2 levels, whereas H3K4me3 is unaltered [37]. Based on our genetic interaction between *lid* and *dKDM2*, this may be because Lid/KDM5a compensates for the loss of dKDM2's H3K4me3 demethylase activity. Conversely, it is likely that dKDM2 also functions outside the nucleolus and that H3K4me3 regulation by Lid and dKDM2 is essential for development. It is important to note, however, that while Lid's demethylase activity is required for the genetic interaction between *lid* and *dKDM2*, we cannot rule out the possibility that dKDM2 requires its H3K36me2 demethylase enzymatic activity not its H3K4me3 activity. Both H3K4me3 and H3K36me2 are chromatin marks associated with active transcription, and it is possible that Lid's H3K4me3 demethylase activity is functionally linked to dKDM2-mediated H3K36me2 demethylation.

### Demethylase-independent functions of the JmjC domain

Among the JmjC domain-containing proteins, Lid is most structurally similar to the founding member of this class of demethylases, Jumonji (JARID2), having a JmjN, ARID and C_5_HC_2_ zinc finger in addition to a JmjC domain. In both mammals and Drosophila, Jumonji is enzymatically inactive because it lacks key residues within its JmjC domain required for Fe^2+^ and α-ketoglutarate binding [38]. Indeed, while Jumonji has been implicated as a regulator of transcription [30], [38]–[40], the molecular function of the JmjC domain has remained elusive. Taken in conjunction with our finding that Lid's demethylase activity is not essential for development, this raises the exciting possibility that the JmjC domain has important demethylase-independent functions. Consistent with this hypothesis, we find that a genomic rescue transgene with a deletion of the JmjC domain fails to rescue *lid* mutants (CG and JS, unpublished). Because more than half the known JmjC domain-containing proteins in mammals and Drosophila do not have an ascribed enzymatic activity, a demethylase-independent functions of this domain may be a common feature of this class of protein.

### PHD finger-mediated histone binding by Lid

Lid has three PHD fingers and we have demonstrated that its N- and C-terminal PHDs bind specific methylated forms of the histone H3 tail. While Lid's N-terminal H3K4me0-binding PHD finger was not required for development, its third PHD finger, which binds to H3K4me2/3, is essential for viability and is required for Lid to function in dMyc-mediated cell growth. One long-standing question regarding many transcription factors is the mechanism by which they find their appropriate binding site within the genome, as many transcription factors recognize short DNA sequences that are similar or identical. This suggests that binding site specificity may additionally involve the recognition of non-DNA elements such as local chromatin environments. In mammalian cells, c-Myc shows a clear binding preference for E boxes located within a chromatin context containing highly di- and trimethylated nucleosomal histone H3K4 [32]. However, the mechanism by which Myc recognizes this chromatin landscape is unclear. We propose that Lid utilizes its H3K4me2/3 binding C-terminal PHD finger to tether Myc to its preferred chromatin context, thereby permitting selection of biologically important E boxes. Further experiments to more precisely define the role of Lid's PHD finger in Myc-mediated cell growth are ongoing.

In summary, we have demonstrated that Lid's JmjC domain-encoded demethylase activity, its histone H3K4me0-binding N-terminal PHD finger and its PHD2 of unknown function, are dispensable for development. In contrast, all other domains of Lid tested were required to rescue *lid* homozygous mutants, including its C-terminal, H3K4me2/3 binding, PHD finger that functions in dMyc-mediated cell growth. These findings highlight the importance of characterizing the function of individual domains of transcriptional regulators such as Lid in order to understand the mechanisms by which they regulate gene expression in a developmental context.

## Materials and Methods

### Fly strains and crosses

UAS-lid and UAS-lidJmjC* have been described previously [7]. All other *Drosophila* strains were obtained from the Bloomington stock center. Deletions within Lid were made in the pUASp vector by site directed mutagenesis and delete the following amino acids: LidΔJmjN (AA160–206), LidΔARID (AA223–314), LidΔPHD1 (AA450–499), LidΔC_5_HC_2_ (AA830–883), LidΔPHD2 (AA1296–1354), LidΔPHD3 (1749–1838 by introducing a stop codon). LidC1296A mutates the first cysteine of Lid's second PHD finger. *lid* genomic rescue transgenes were generated by fusing a 4.5 kb PCR-generated Xho I fragment containing the *lid* upstream region and a 4.8 kb Xho I/Not I fragment containing the remainder of the *lid* coding sequence (either wildtype or JmjC^*^) into the vector pCasper4. All transgenic flies were generated by The Best Gene (thebestgene.com). Lifespan studies were carried out as described by [41].

To test the ability of UAS-Lid (wildtype and deletion) transgenes to rescue the *lid* mutant phenotype, a UAS-Lid (or deletion) transgene was recombined onto the *lid^10424^* chromosome. At least 2 independent P element insertions were tested for each to minimize chromosomal position effects. This *lid^10424^*, UAS-Lid (or deletion) recombinant chromosome, balanced over CyO, was then mated to the *lid^10424^*/CyO; Actin-Gal4/TM6B strain. Rescue was assessed by scoring the presence of straight winged, non-TM6B, progeny. Somatic clones overexpressing UAS transgenes marked by the co-expression of GFP were generated as described in [42]. Longevity studies were carried out as described in [41] and paraquat assays as described in [43].

### In vitro binding assays

Histone binding assays: 1 µg of biotinylated histone peptides (Fisher) were incubated with 5 µg purified GST-PHD finger in 1 ml of binding buffer (50 mM Tris pH 7.5, 200 mM NaCl, 2 mM dithiothreitol, 0.5% Nonidet P-40 (v/v), 1 µM ZnSO_4_, 1% BSA) at 4°C overnight. Complexes were then immobilized using 10 µl Streptavidin-agarose beads (Invitrogen) for 1 hr at 4°C. Immobilized complexes were then washed three times with 1 ml of binding buffer, boiled and loaded on a 4–12% gel. Gels were stained with coomassie blue to visualize bound GST-PHD protein. GST-protein binding assays: 1 µg of purified GST-dMycC [7] was incubated with S^35^-labeled Lid or Lid deletion proteins made using rabbit reticulocyte lysate (Invitrogen) in 1xPBS, 1% BSA and 0.5%NP-40, washed in 1xPBS, 0.5% NP-40, boiled and loaded onto a 4–12% protein gel. GST-dMycC was visualized using coomassie brilliant blue and S^35^ detected via standard procedures.

### Antibodies, Westerns, and immunofluorescence

The Lid rabbit and dMyc antibodies have been described previously [7], [44]. Anti-trimethylated H3K4 and H3K36me2 were obtained from Active Motif, and γ-tubulin from Sigma. Western analysis was carried out using standard protocols, infrared conjugated secondary antibodies (LiCOR) and Odyssey scanner and software. Immunofluorescence was carried out as described in [7]. Quantitation of Western blots was carried out using LiCOR odyssey v3.0 software.
